# Detecting drug-resistant tuberculosis in chest radiographs

**DOI:** 10.1007/s11548-018-1857-9

**Published:** 2018-10-03

**Authors:** Stefan Jaeger, Octavio H. Juarez-Espinosa, Sema Candemir, Mahdieh Poostchi, Feng Yang, Lewis Kim, Meng Ding, Les R. Folio, Sameer Antani, Andrei Gabrielian, Darrell Hurt, Alex Rosenthal, George Thoma

**Affiliations:** 1 Lister Hill National Center for Biomedical Communications, U.S. National Library of Medicine, Bethesda, MD 20894 USA; 2 Office of Cyber Infrastructure and Computational Biology, National Institute of Allergy and Infectious Diseases, Rockville, MD 20852 USA; 3 Radiology and Imaging Sciences, Clinical Center, National Institutes of Health, Bethesda, MD 20892 USA; 4 School of Computer and Information Technology, Beijing Jiaotong University, Beijing, 100044 China; 5 Bayer HealthCare, 1 Bayer Dr, Indianola, PA 15051 USA

**Keywords:** Biomedical imaging, Machine learning, Computer-aided diagnosis, Tuberculosis, Drug resistance

## Abstract

**Purpose:**

Tuberculosis is a major global health threat claiming millions of lives each year. While the total number of tuberculosis cases has been decreasing over the last years, the rise of drug-resistant tuberculosis has reduced the chance of controlling the disease. The purpose is to implement a timely diagnosis of drug-resistant tuberculosis, which is essential to administering adequate treatment regimens and stopping the further transmission of drug-resistant tuberculosis.

**Methods:**

A main tool for diagnosing tuberculosis is the conventional chest X-ray. We are investigating the possibility of discriminating automatically between drug-resistant and drug-sensitive tuberculosis in chest X-rays by means of image analysis and machine learning methods.

**Results:**

For discriminating between drug-sensitive and drug-resistant tuberculosis, we achieve an area under the receiver operating characteristic curve (AUC) of up to 66%, using an artificial neural network in combination with a set of shape and texture features. We did not observe any significant difference in the results when including follow-up X-rays for each patient.

**Conclusion:**

Our results suggest that a chest X-ray contains information about the likelihood of a drug-resistant tuberculosis infection, which can be exploited computationally. We therefore suggest to repeat the experiments of our pilot study on a larger set of chest X-rays.

## Introduction

Tuberculosis (TB) is a serious worldwide public health threat [1]. It is an airborne disease that is caused by mycobacterium tuberculosis (MTB) bacteria, which was first discovered in 1882 [2]. The worldwide TB mortality rate is decreasing due to a global effort to improve TB control and treatment. However, even today after the development of advanced medical treatment and diagnostic technology, TB is the leading cause of death from infectious disease in the world. About 9.6 million people are estimated to have TB, which claimed 1.5 million lives in 2014 alone.

Particularly, worrisome are the drug-resistant forms of tuberculosis. Multidrug-resistant TB (MDR-TB) is a form of tuberculosis that is resistant to treatment with one or two of the first-line anti-TB drugs (isoniazid and rifampicin). MDR-TB is concerning because it is difficult to diagnose and it takes more time (often more than two years) and cost to treat patients. About 3.3% of new TB cases and 20% of previously treated cases are estimated to have MDR-TB. Globally, the trend of drug-resistant tuberculosis has remained unchanged at best [3].

One of the major challenges for controlling MDR-TB lies in the difficulty in diagnosing drug resistance of TB-suspected patients during their first visit. Conventionally, drug susceptibility testing is performed on a sputum sample to identify the resistant status to several drugs, which requires a well-equipped laboratory facility and takes four to six weeks to obtain the laboratory results. The recent development of the Xpert MTB/RIF, a real-time test based on polymerase chain reaction (PCR) for genetic mutations in the MTB genome associated with resistance, specifically rifampicin (RIF) resistance, has greatly reduced the laboratory time needed for the detection of MDR-TB [1]. However, the test still produces a large number of inconclusive results and its deployment in resource-constrained settings is expensive. In addition, the test requires a sputum sample, which can be difficult to obtain, especially from children. Therefore, detecting MDR-TB is still a challenge and the conventional chest CXR (X-ray) remains a valuable tool in detection, screening and surveillance of MDR-TB, thanks to its widespread availability.

With the recent advances in imaging technology and computational methods for quantitative imaging, infectious disease imaging (IDI) has shown promising results in infectious disease diagnosis. IDI is an interdisciplinary field involving clinical research of infectious diseases under various imaging modalities, including CXR, computed tomography (CT), positron emission tomography (PET), magnetic resonance imaging (MRI), and other modalities. One goal is to leverage radiology for the diagnosis and treatment of emerging pathogens, epidemics and pandemics. Clinical imaging allows obtaining quantitative information and applying computer-assisted detection methods for assessing infectious disease severity and response to therapy. IDI, therefore, offers new ways of diagnosing infectious diseases effectively and accurately. For example, in H1N1 influenza diagnosis, computed tomography (CT) imaging of severe H1N1 contributed to earlier diagnosis and treatment of the infection by eliminating other possible causes of disease. For monitoring tuberculosis treatment, PET/CT scans were computationally evaluated and provided a volumetric assessment of TB-associated abnormalities, which were predictive of treatment outcomes [4].

We have structured the paper as follows: Background and previous work section describes the background of our work on discovering potential radiological features that could indicate drug resistance. Third section provides information about the set of CXRs we have acquired for our studies and presents our methods for lung segmentation, feature computation, and classification. Finally, fourth section shows our results, followed by a final conclusion summarizing the paper.

## Background and previous work

### Drug resistance

Tuberculosis is a curable infection. However, it requires a long treatment with several drugs (www.tbfacts.org). There are currently more than 20 drugs in use against TB. The five most commonly used drugs, which are also called first-line drugs, are typically used for TB patients without prior TB drug treatment. This includes the drugs isoniazid, rifampicin, and three others. It is essential that several TB drugs are being taken together to avoid becoming resistant to an individual drug. To avoid drug resistance, it is also very important that the patient adheres strictly to the treatment regimen over several months without interruptions.

The drugs for the treatment of drug-resistant TB are more expensive and have more side effects. They are grouped according to their effectiveness and experience of use, and belong to the so-called second-line drugs, which are the reserve drugs for treating drug resistance.

A patient with TB is drug-susceptible if the TB bacteria causing the infection respond to all drugs. If a patient has contracted drug-resistant TB, either from the direct transmission from another infected person or due to improper treatment, the TB bacteria will not respond to at least one of the main drugs. Two main types of drug resistance are MDR-TB and XDR-TB. MDR-TB, or multidrug resistant TB, is defined as resistance to at least isoniazid or rifampicin, which are two of the most effective first-line TB drugs. XDR-TB, or extensively drug-resistant TB, is caused by bacteria that, in addition to resistance against isoniazid or rifampicin, are resistant to additional drugs, including at least one of the second-line drugs. These are the two main types of TB drug resistance, although additional categories are sometimes used depending on the number of drugs that the bacteria do not respond to, including resistance against individual drugs and resistance against most of the existing drugs. Treatment of the latter is extremely difficult.

According to the latest WHO update on MDR-TB, there were an estimated 480,000 new cases of MDR-TB in 2015 [3]. Almost 10% of these cases are extensively drug-resistant (XDR-TB). To date, 117 countries have reported at least one XDR-TB case.

### Previous work

There is evidence that differentiating between MDR-TB and drug-sensitive TB may be possible in computed tomography (CT). For example, Yeom et al. [5] found significant correlation of bilateral and multiple findings such as segmental or lobar consolidation and cavities with primary MDR-TB patients. Findings such as bilateral consolidations and multiple cavities were also evident in the CXRs of our MDR-TB patients, making them an ideal cohort for discrimination analysis (see “Methods and procedures” section).

Chen et al. [4] correlated PET/CT imaging with treatment outcome in patients with multidrug-resistant TB. They assessed changes at 2 and 6 months (CT only) in a cohort of 28 subjects with multidrug-resistant TB, who were treated with second-line TB therapy for 2 years and then followed for an additional 6 months. CT scans were read semiquantitatively by radiologists and were computationally evaluated using custom software to provide volumetric assessment of TB-associated abnormalities. Their results show that CT scans at 6 months (but not 2 months) assessed by radiologist readers were predictive of outcomes, and changes in computed abnormal volumes were predictive of drug response at both time points. In their cohort, some radiologic markers were more sensitive than conventional sputum microbiology in distinguishing successful from unsuccessful treatment. While these results support the potential of imaging scans, the authors admit that larger cohorts confirming these results are needed.

An early study by Cha et al. [6] was designed to describe radiological findings of XDR-TB and to compare the observed findings with the findings of drug-sensitive and MDR-TB in non-AIDS patients. Their conclusion was that by observation of multiple cavities, nodules, and bronchial dilatation as depicted in CT in young patients, the presence of MDR-TB or XDR-TB rather than drug-sensitive TB can be suggested. There was no significant difference in imaging findings between patients with XDR-TB and MDR-TB.

The result of Cha et al. confirmed the result of an even earlier study by Kim et al. [7], who observed that patients with MDR-TB had visible cavity formations on CT and concluded that multiple cavities suggest the possibility of MDR-TB. This is also consistent with a study by Chung et al. [8]. However, Lee et al. [9] concluded later that CT findings of XDR-TB are indeed similar to those of MDR-TB, but XDR-TB tends to have more extensive consolidation and tree-in-bud appearance.

Very little work has been done to discriminate between drug-sensitive and drug-resistant TB automatically by computational means, let alone achieving this for the common CXR. In an early pilot study, Kovalev et al. [10] observed statistically significant links between computerized features of radiological images and drug resistance status of TB patients. In a second study, the authors achieved an accuracy of more than 75% but only when combining CXR with CT features [11]. The performance for CXR features alone was much lower.

## Methods and procedures

We process CXR images through a pipeline consisting of lung segmentation, feature computation, and classification.

### Image acquisition and annotation

For our study, we are using CXR images from a patient database of the Republic of Belarus, where MDR/XDR-TB and HIV/TB are prevalent. In addition to the CXR images of all patients, the database includes laboratory work and clinical data. All images have been collected as part of Belarus’ compulsory lung screening program, in which the population is regularly screened for lung diseases. All patients had been admitted to the MDR-TB department of the Republic’s Scientific and Practical Center of Pulmonology and Tuberculosis (RSPCPT) with either already diagnosed or suspected MDR-TB. Each patient received a radiological examination not long after the date of registration. For image acquisition, the KODAK Point of Care 260 CR System with the KODAK Quality Control Software (Version 2.1.2.0.) has been used.

The 135 cases investigated in this paper consist of 45% sensitive (61) amd 54% MDR (74) cases. The gender distribution is 59% males (80) and 40% females (55). Among these patients, 61% (83) are younger than 50 years and 39% (52) are older than 50 years. Table 1 lists the data stratified according to age, gender, and type of resistance.

**Table 1:** Exp. 1—Belarus CXRs stratified by age, gender, and type of resistance

Age	Sensitive		MDR	
	Male	Female	Male	Female
$< 50$	22	15	26	20
$\ge 50$	12	12	20	8

The data given in Table 1 will be the basis for our first experiment later in the paper, in which we use the initial CXR of each patient to discriminate between sensitive TB and MDR-TB. For these data, we measured no significant difference between the age and gender distributions among sensitive and resistant TB (*z*-test, with $p=0.57$ and $p=0.77$, respectively).

In our second experiment, we include the follow-up CXRs for each patient. Table 2 lists the number of CXRs of the second experiment stratified according to age, gender, and type of resistance. For the data of the second experiment, we measured again no significant difference between the age and gender distributions among sensitive and resistant TB (*z*-test, with $p=0.19$ and $p=0.71$, respectively).

**Table 2:** Exp. 2—Belarus CXRs and follow-ups stratified by age, gender, and type of resistance

Age	Sensitive		MDR	
	Male	Female	Male	Female
$< 50$	72	36	62	47
$\ge 50$	22	27	45	16

Patients who had follow-up visits took additional CXRs during the visits. Sensitive patients had about four CXRs on average, and MDR-TB patients had an average of roughly three CXRs taken in comparison, see Table 3.

**Table 3:** Number of CXRs per patient

	Sensitive	MDR
Maximum number of CXRs	23	17
Minimum number of CXRs	1	1
Mean	3.9	2.6
SD	3.5	2.3

The time interval between CXRs is given in Table 4 as days between follow-up visits. On average, the sensitive TB patients had about 45-day intervals between visits and the MDR-TB patients had an average of 38 days between visits. Follow-up times vary depending on patients.

**Table 4:** Time gaps between CXRs

Gap	Sensitive (days)	MDR (days)
Mean	44.5	38.4
SD	39.9	26.9
Min	1	1
Max	146	139

The sensitive TB patients received the standard WHO recommended TB regimens: ethambutol (e), isoniazid (h), rifampicin (r), and pyrazinamide (z) (Table 5).

**Table 5:** Frequency of treatment regimens among sensitive TB cases

Treatment regimen	Frequency
Ethambutol (e)	61
Isoniazid (h)	61
Rifampicin (r)	61
Pyrazinamide (z)	61

For the MDR-TB patients, treatment regimens were prescribed based on drug-resistant tests in a microbiology laboratory. Therefore, each MDR-TB patient’s prescription differs and is a combination of several TB drugs, excluding regimens that were resistant. Frequently prescribed treatments were capreomycin (cm), cycloserine (cs), levofloxacin (lfx), p-aminosalicylic acid (pas), protionamide (pto), and pyrazinamide (z) (Table 6). Most of these patients were resistant to ethambutol (e), isoniazid (h), and rifampicin (r) of the standard TB regimen.

**Table 6:** Frequency of treatment regimens among MDR-TB patients

Treatment regimen	Frequency
Amikacin (am)	11
Amoxicillin/clavulanate (amx_clv)	7
Capreomycin (cm)	52
Cotrimoxazol (c)	2
Cycloserine (cs)	70
Ethambutol (e)	7
Isoniazid (h)	2
Kanamycin (km)	9
Levofloxacin (lfx)	60
Linezolid (lzd)	1
Moxifloxacin (mfx)	3
Ofloxacin (ofx)	9
p-aminosalicylic acid (pas)	63
Protionamide (pto)	71
Rifampicin (r)	2
Pyrazinamide (z)	53

### Lung segmentation

**Fig. 1:**
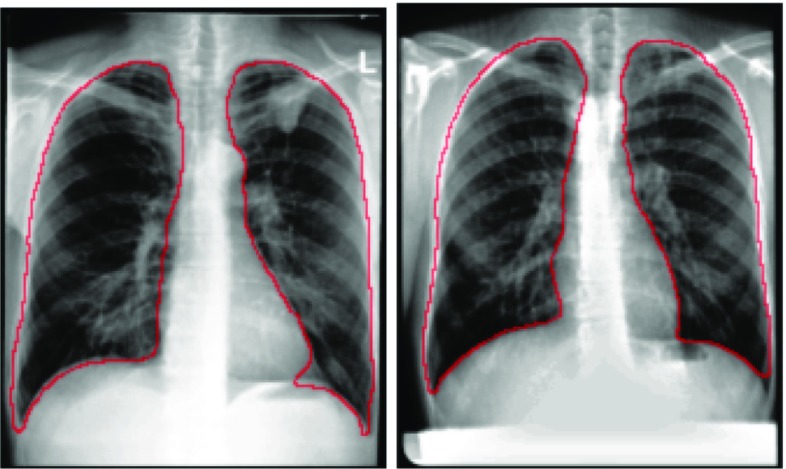
Two CXRs with their detected lung boundaries

We detect lung boundaries in our CXRs using an atlas-based lung segmentation algorithm [12]. The algorithm uses existing CXRs and their manually delineated lung boundaries as models and estimates the unknown lung boundary of a patient’s CXR by registering the existing models to the patient’s CXR. As models, we use our public CXR dataset with reference lung boundaries [12, 13]. For a patient CXR, the algorithm first finds the most similar CXRs in the model set. We measure the similarity between CXRs by comparing the horizontal and vertical intensity histograms, which serve as rough shape descriptors of the lung, using the Bhattacharyya distance as a similarity measure. The main purpose of measuring the similarity is to limit the actual registration to the most similar models in order to reduce computational costs. After the model selection, we compute the correspondences between the model CXRs and the patient CXR. To do so, we create a correspondence map by first describing the patient CXR using local image features and then finding the most similar locations with a matching algorithm, following the SIFT flow approach [14]. The SIFT flow algorithm models local gradient information of the observed image using the scale-invariant feature transform (SIFT) [15]. Once we have calculated the SIFT features, the registration algorithm computes pixel-to-pixel correspondences by matching the SIFT features. The computed map of corresponding pixels between images then serves as a transformation matrix that we use to generate the approximate lung model for the patient CXR. We have shown that this algorithm produces state-of-the-art results for lung boundary detection on the public JSRT set [16], among others. For more details about the algorithm, we refer to [12]. Figure 1 shows two examples of lung boundaries detected by our method.

### Feature computation

For our classification experiments, we use two different feature sets as described in the following. The first set is based on color, shape, and texture, whereas the second set is based on edge orientation.

*Color, shape, and texture features* To describe visual patterns indicating drug resistance or drug sensitivity in a segmented lung field, we use a feature set that we have successfully used in [17] to discriminate between TB-infected and uninfected lungs. It is a combination of shape, edge, and texture descriptors [18]. For each descriptor, we compute a histogram that shows the distribution of the different descriptor values across the lung field. Each histogram bin serves as a feature, and all features of all descriptors combined represent the feature vector input to a classifier. The following is a list of all descriptors we are using, with each descriptor quantized into 32 bins (see also [19–21]):

- Intensity histograms (IH)
- Gradient magnitude histograms (GM)
- Shape descriptor histograms (SD)  [22]
  1 $$\begin{aligned} \hbox {SD} = \tan ^{-1}\left( \frac{\lambda _1}{\lambda _2}\right) , \end{aligned}$$
  where $\lambda _1$ and $\lambda _2$ are the eigenvalues of the Hessian matrix, with $\lambda _1 \le \lambda _2$.
- Curvature descriptor histograms (CD) [22]
  2 $$\begin{aligned} \hbox {CD} = \tan ^{-1}\left( \frac{\sqrt{\lambda _1^2 + \lambda _2^2}}{1+I(x,y)}\right) , \end{aligned}$$
  with $0 \le \hbox {CD} \le \pi /2$, where *I*(*x*, *y*) denotes the pixel intensity for pixel (*x*, *y*). The normalization with respect to intensity makes this descriptor independent of image brightness.
- Histogram of oriented gradients (HoG) is a descriptor for gradient orientations weighted according to gradient magnitude [22, 23].
- Local binary patterns (LBP) are texture descriptors that code the intensity differences between neighboring pixels by a histogram of binary patterns, which are generated by thresholding the relative intensity between all pixels and their neighboring pixels [24, 25]. LBP are among the most successful features in image analysis and often used in combination with HoG [19, 20, 22].

With these six descriptors, our overall number of features is $6*32=192$. The eigenvalues of the Hessian matrix needed for the shape and curvature descriptors in Eqs. 1 and  2 were computed using a modification of the multiscale approach by Frangi et al. [26, 27]. We first smooth the lung region using a Gaussian filter *G*(*x*, *s*) at each pixel *x* and different scales *s* ($s=2, 4, 6,\ldots , 20$) and then compute the eigenvalues of the Hessian matrix on different scales, which captures the second-order characteristics.

To capture the visual features of drug resistance, we implement the following filter *F* that responds to spherical shapes, using the Hessian eigenvalues $\lambda _1$ and $\lambda _2$:

3 $$\begin{aligned} F = 1-e^{-\sqrt{k\cdot |\lambda _1*\lambda _2|}}. \end{aligned}$$

The larger the eigenvalues, the larger the filter response. We use the eigenvalues of the scale resulting in the largest response to compute the shape and curvature descriptors above [17].

**Fig. 2:**
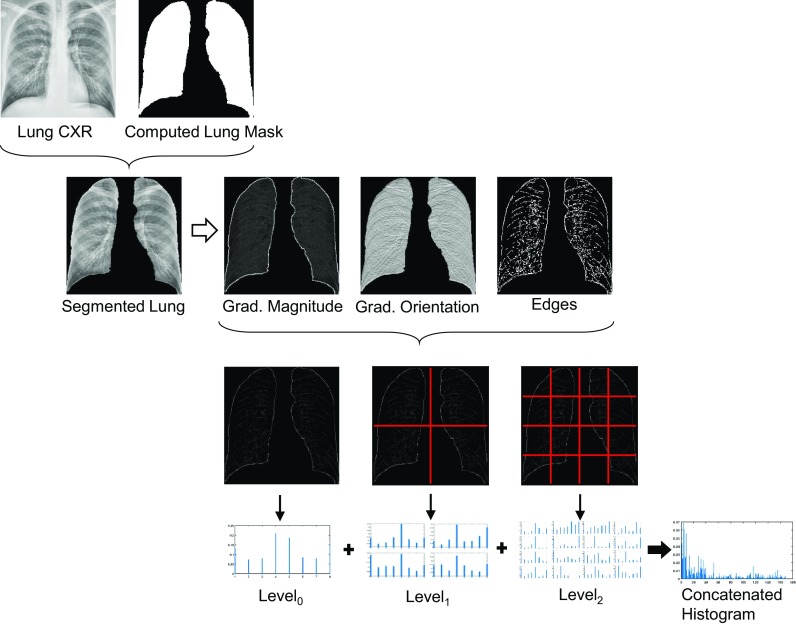
PHoG feature computation for CXR

We also use Pyramid Histogram of Oriented Gradients (PHoG), which is a popular region descriptor in many object recognition systems [28–31]. PHoG represents the region by its local shape and spatial layout of the shape. Spatial layout is preserved by tiling the image into small patches at multiple resolutions. Figure 2 illustrates three-level PHoG computations for a sample lung CXR image.

Each candidate region is divided into a finer spatial grid like a quad-tree. At the lowest level of the pyramid ($L_{0}$), a coarse histogram descriptor encodes the entire region, while finer-region grids are covered at higher-resolution pyramid levels. The dimension of the concatenated histogram is $K\sum _{l=0}^{L}4^{l}$, where *K* is the number of bins and *L* is the number of pyramid levels. For our experiments, we computed a three-level spatial pyramid of HoG with eight bins leading to a feature vector of dimension 680.

### Classification

To discriminate between drug-resistant and drug-sensitive TB, we test different classifier architectures, which we list in the following.

*Support vector machine* We use a binary linear support vector machine (SVM), which classifies the computed feature vectors into one of the natural two classes, sensitive or resistant [32, 33]. The SVM computes the best separating hyperplane, which is the hyperplane with the largest distance to the nearest training data point of any class, between the feature vectors of both classes, as presented in a training set. The advantage of an SVM classifier compared to other recent classification methods, such as deep learning in particular, is that it can achieve a very good performance when trained on a small training set. It does not need as many training samples as required by deep learning for example. Mathematically, the majority of the feature vectors representing lungs with manifestations of drug resistance will be on the one side of the hyperplane, while feature vectors for lungs with drug-sensitive TB will be on the other side. Therefore, we are using the signed distance to the hyperplane as our confidence in a lung showing signs of drug resistance.

*Artificial neural network (ANN)* We used MATLAB standard pattern recognition neural network which is a two-layer feedforward network with a sigmoid transfer function in the hidden layer and a softmax transfer function in the output layer. The number of hidden neurons is comparable with the feature vector size.

*Deep learning* Deep learning has recently become a very popular classification scheme in medical applications and computer-aided diagnosis (CAD) in particular [34, 35]. It usually requires very large training sets but can often outperform traditional classification approaches [36]. It is an extension of traditional artificial neural networks in that networks typically have deeper structures with different types of network layers. We run deep learning experiments with both a pre-trained network and a customized network.

**Fig. 3:**
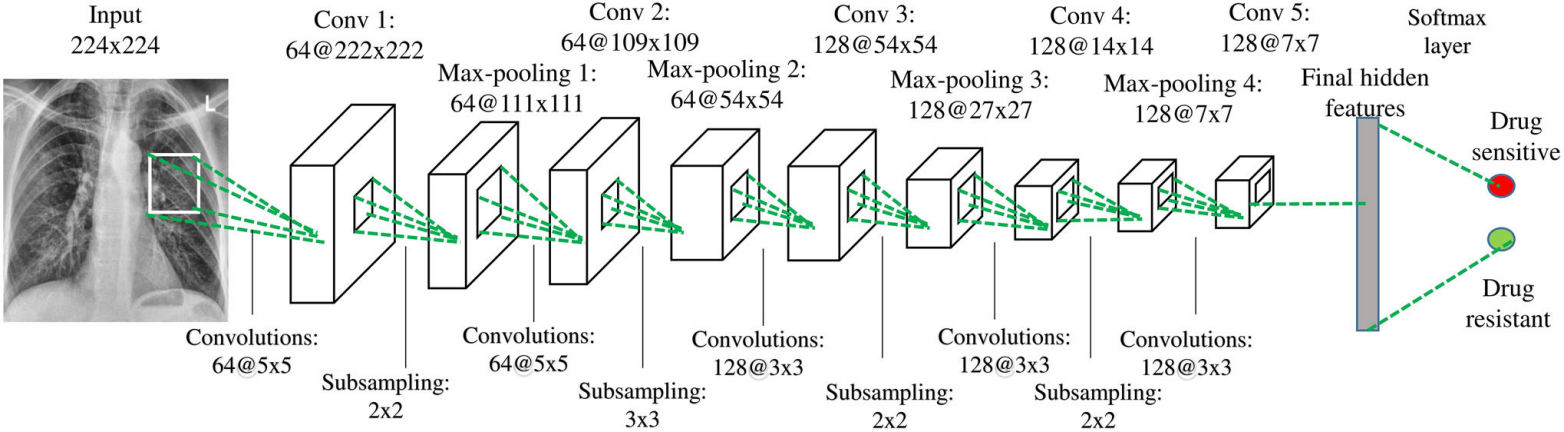
Our customized CNN architecture for chest CXR classification. The numbers above the cuboid indicate the dimensions of the feature maps. The numbers below the green dotted lines represent the convolutional kernel size and the size of the max-pooling region. The output layer is a softmax layer that predicts the probability of drug sensitivity

- VGG-v16 For our pre-trained network, we use the VGG-v16 network architecture, which was introduced by Simonyan and Zisserman in 2014 for image classification [37]. It is characterized by its simplicity, using only 33 convolutional layers stacked on top of each other in increasing depth. Reducing volume size is handled by max pooling. Two fully connected layers, each with 4096 nodes, are then followed by a softmax classifier. The VGG-v16 has been pre-trained on more than a million images and has therefore learned rich feature representations for a wide range of images. We train this network and adapt it to our data, which is also called transfer learning.
- CNN For our customized network, we follow a general network architecture for convolutional neural networks. Our network starts from a convolutional layer, which convolves the input feature maps with a number of convolutional kernels and yields a corresponding number of output feature maps. In order to perform a nonlinear transformation from the input to the output space, we adopt the rectified linear unit (ReLu) nonlinearity for each convolution [38]. Following each convolutional layer, a max-pooling layer is introduced to select feature subsets. The last convolutional feature map is connected to two fully connected layers with 512 and 2 hidden units, respectively. Between the two fully connected layers, we use a dropout layer with a dropout ratio of 0.4 to reduce overfitting (Fig. 3).

## Results

In our first experiment, we test our classifiers on the 135 patients from Belarus, including 61 CXRs from patients with drug-sensitive TB and 74 CXRs from patients with MDR-TB and no follow-up CXRs.

**Table 7:** Area under the ROC curve (AUC) computed for six different classification methods using fivefold cross-evaluation

	Experiment 1 (%)	Experiment 2 (%)
ANN_Shape_Texture_Fts	65	66
CNN	56	62
SVM_Shape_Texture_Fts	57	58
ANN_PHoG	55	59
SVM_PHoG	50	61
VGG-v16	52	57

In particular, we list the AUC for six different classifiers, namely the ANN with our shape and texture features, our customized CNN, SVM with shape and texture features, ANN with PHoG, SVM with PHoG, and a classifier based on a pre-trained VGG-v16 network (Table 7). The ANN with shape and texture features shows the best performance on Experiment 1, with an AUC of $65\%$, whereas the other classifiers provide a much lower performance. The traditional ANN outperforms the customized CNN network, which we attribute to the small training set, which has been a known problem of large deep learning networks with many variables.

**Table 8:** Accuracy and $F_\mathrm{measure}$ for the ANN classifier and fivefold cross-evaluation

	Experiment one						Experiment two					
	TP	FP	TN	FN	Accuracy	$F_\mathrm{measure}$	TP	FP	TN	FN	Accuracy	$F_\mathrm{measure}$
Fold 1	14	1	7	5	0.78	0.82	29	12	19	8	0.71	0.74
Fold 2	7	8	6	6	0.48	0.50	19	15	17	10	0.59	0.60
Fold 3	6	8	7	6	0.48	0.46	13	17	33	8	0.65	0.51
Fold 4	10	5	9	3	0.70	0.71	23	11	19	17	0.60	0.62
Fold 5	9	6	6	6	0.56	0.60	16	15	16	10	0.56	0.56
Total/avg	46	28	35	26	0.60 (avg)	0.62 (avg)	100	70	104	53	0.62 (avg)	0.61 (avg)

Table 8 shows the accuracy and $F_\mathrm{measure}$ for the ANN classifier trained with shape and texture features based on fivefold cross-evaluation. The results for Experiment 1 are on the left-hand side of Table 8. For each fold, Table 8 reports the number of true positives (TP), false positives (FP), true negatives (TN), false negatives (FN), accuracy, and $F_\mathrm{measure}$, where accuracy and $F_\mathrm{measure}$ are computed as follows:

4 $$\begin{aligned} \hbox {Accuracy}= & {} \frac{\mathrm{TP} + \mathrm{TN}}{\mathrm{TP}+\mathrm{FP}+\mathrm{TN}+\mathrm{FN}}, \end{aligned}$$

5 $$\begin{aligned} F_\mathrm{measure}= & {} 2\times \frac{\mathrm{Precision} \times \mathrm{Recall}}{\mathrm{Precision} + \mathrm{Recall}}, \end{aligned}$$

6 $$\begin{aligned} \hbox {Precision}= & {} \frac{\mathrm{TP}}{\mathrm{TP}+\mathrm{FP}}, \quad \hbox {Recall} = \frac{\mathrm{TP}}{\mathrm{TP}+\mathrm{FN}}. \end{aligned}$$

According to Table 8, we achieve an average accuracy of $60\%$ and an average $F_\mathrm{measure}$ of $62\%$ for the ANN classifier, with shape and texture features, for Experiment 1.

**Fig. 4:**
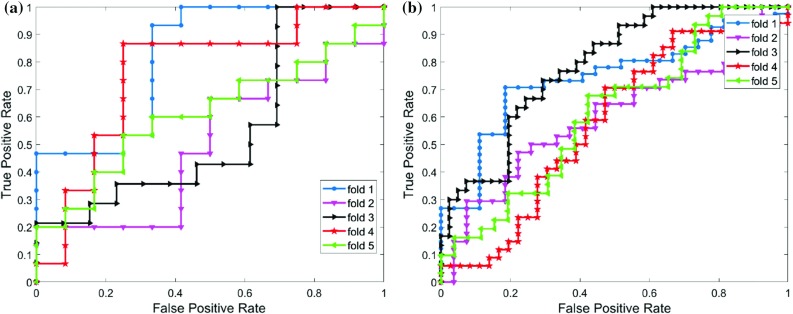
ROC curves for drug-sensitive TB vs MDR-TB classification without (left) and with follow-up CXRs (right) using ANN with shape and texture features

The left-hand side of Fig. 4 shows our classification performance for the first experiment, measured as the area under the receiver operating characteristic curve (AUC) for the ANN classifier with shape and texture features. Due to the smaller training and test set size in Experiment 1, the ROC curves in Fig. 4a have a staircase shape and vary largely. For Experiment 1, Table 9 lists the results of the ANN classifier on shape and texture features, including AUC and accuracy (ACC), separated according to gender and age.

In a second experiment, we add the follow-up CXRs for all patients to the set, which increases the number of CXRs to 327, including 157 CXRs with drug-sensitive TB and 170 CXRs with drug-resistant TB (MDR). Table 2 lists the numbers of CXRs stratified according to age, gender, and resistance for Experiment 2. The right-hand side of Table 7 shows the AUC results of each of our classifiers for Experiment 2. Again, the ANN with shape and texture features performs the best, with an AUC of $66\%$. This result differs only little from the performance in the first experiment, indicating that including follow-up CXRs does not add more information for discriminating between sensitive and MDR-TB.

On the right-hand side of Table 8, we list the accuracy and $F_\mathrm{measure}$ for the ANN classifier trained with shape and texture features and fivefold cross-evaluation in Experiment 2. Similar to Table 7, the results are very close to the results observed for Experiment 1.

On the right-hand side of Fig. 4, we show the ROC curves for the ANN classifier for each fold in the second experiment. Compared to the curves on the left-hand side from Experiment 1, the variance is much lower due to the larger training set including follow-up CXRs.

Finally, Table 10 presents the AUC and accuracy for the ANN classifier trained with shape and texture features in Experiment 2, and stratified according to gender and age.

## Conclusion

We investigate the possibility of using the conventional CXR to discriminate between drug-sensitive and drug-resistant forms of TB. For our experiments, we use different classifiers and features, including shape and texture features that have provided good results for computer-aided TB screening in CXRs, which assures us that the features we use can pick up TB-relevant textures and shapes in the lung field.

**Table 9:** ANN performance evaluation using texture and shape features for Experiment 1, with data stratified according to age and gender

	#images	AUC (%)	ACC (%)
Exp. 1 (ANN $+$ features)			
Female	55	60	58
Male	80	63	56
$\hbox {Age}<50$	83	61	55
$\hbox {Age}\ge 50$	52	71	66

**Table 10:** ANN performance evaluation using texture and shape features for Experiment 2, with data stratified according to age and gender

	#images	AUC (%)	ACC (%)
Exp. 2 (ANN $+$ features)			
Female	126	61	55
Male	201	71	66
$\hbox {Age}<50$	217	68	60
$\hbox {Age}\ge 50$	110	57	55

We perform two experiments: The first experiment includes only the initial CXR of each patient, whereas in the second experiment, we also include the follow-up CXRs for each patient. For both experiments, we train our classifiers and evaluated them by fivefold cross-evaluation. As features, we use our established set of texture and shape features and the PHoG descriptor. The best performance is achieved by a traditional neural network classifier (ANN) in combination with shape and texture features, which results in an AUC of $65\%$ for the first experiment and $66\%$ for the second experiment. We also experimented with two deep learning networks, a pre-trained VGG-v16 network and a customized convolutional neural network (CNN). Both networks do not perform better than our ANN. We attribute this to our relatively small training set size, which poses a problem for deep learning networks with many variables.

For both experiments, we stratified the data according to gender and age and present the corresponding AUC values and accuracies of our best performing ANN classifier.

Our approach relies solely on 2D CXR features and is in line with the few publications in the literature that can only report higher performance when including 3D information from CTs. In summary, our experiments provide evidence that automatic discrimination between drug-sensitive and drug-resistant TB can be possible in the conventional chest CXR. Generally speaking, automated screening for drug resistance in radiographs is still an open problem. Our results indicate that CXRs contain information about drug resistance. Describing the nature of this information will be the subject of future research. We can already say that discriminating between drug-resistant TB and drug-sensitive TB based on a CXR alone is difficult, and a much harder problem than detecting TB for which we obtain higher performance values. To obtain better results, it is therefore very promising to repeat this pilot study on larger training sets.
